# Supplementary material for: Thermodynamically consistent machine learning model for excess Gibbs energy
Source: Nat Commun. 2026 Apr 14;17:3485. doi: 10.1038/s41467-026-71430-y (PMC13079877; doi:10.1038/s41467-026-71430-y)
Supplement: Supplementary file 1 — Supplementary Information [file 41467_2026_71430_MOESM1_ESM.pdf]

Supplementary Information to Thermodynamically consistent machine learning model for excess Gibbs energy

Marco Hoffmann[1†], Thomas Specht[1†], Quirin Göttl[2], Jakob Burger[2], Stephan Mandt[3], Hans Hasse[1], Fabian Jirasek[1*]

[1]Laboratory of Engineering Thermodynamics, RPTU Kaiserslautern, Kaiserslautern, Germany.

[2]Laboratory of Chemical Process Engineering, Technical University of Munich, Munich, Germany.

[3]Department of Computer Science & Statistics, University of California, Irvine, Irvine, CA, USA.

*Corresponding author(s). E-mail(s): fabian.jirasek@rptu.de;
Contributing authors: marco.hoffmann@rptu.de; thomas.specht@rptu.de; qgctvtum@gmail.com; burger@tum.de; mandt@uci.edu; hans.hasse@rptu.de;
†These authors contributed equally to this work.

Suppl. Note 1. Surrogate solver

Over all folds and test systems we find that the phase compositions x_i', x_i'' are predicted with an average error of about 0.004, which is likely lower than the uncertainties of most experimental data points. In Suppl. Fig. 1 we provide an example of the functionality of the surrogate solver on a test system of the first fold, namely the system hexane + water. The example demonstrates that, for this system, the surrogate solver's predictions perfectly match the CEM's calculations and that the two methods can be used interchangeably.

Suppl. Note 2. Grid search

The optimal hyperparameters of HANNA were determined using a grid search performed on the first fold. In this work, we varied only the weights of the TPX loss, LLE loss, and HE loss, cf. Eqs. (9), (11), (13) in the manuscript, but not additional hyperparameters of the HANNA architecture, such as the number of nodes per layer or the number of layers in the FFNNs, which were adopted from our prior work [1]. The values for the Gibbs loss and the Lipschitz regularization, cf. Eqs (15) and (20) in the manuscript, were determined in preliminary studies and not changed in the gridsearch. Suppl. Tab. 1 shows the search space and marks the hyperparameters of the selected model configuration. The search space also includes model variants trained without TPX, LLE, or HE data (by setting the respective weights to zero) to assess their general impact on model performance. To improve the robustness of the evaluation, for each hyperparameter setting, an ensemble of two models with different initial weights was trained on the training data of fold one, and their predictions were averaged. The hyperparameter selection was based on the error scores across different data types on the validation set of fold 1, as well as on the percentage of LLE systems for which the

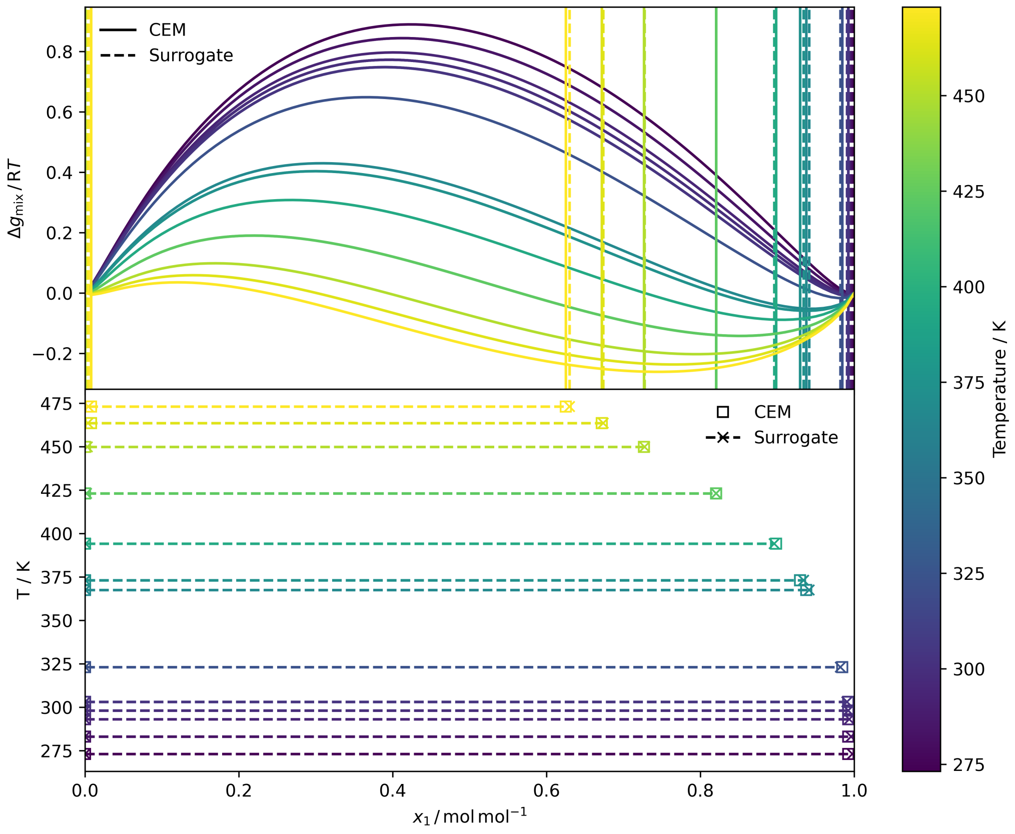

Supplementary Figure 1: Evaluation of the surrogate solver of fold one on the test system hexane (1) + water (2). Top: Gibbs energy of mixing $\Delta g_{\text{mix}}/\text{R}T$ curves over the mole fraction x_1 calculated from mod. UNIFAC for different temperatures. The vertical lines show the LLE phase compositions calculated by the CEM (solid) and predicted by the surrogate solver (dashed). Bottom: LLE phase diagram for the system obtained with the CEM and the surrogate solver. Colors in both diagrams correspond to the temperatures indicated by the color bar.

model correctly predicted a liquid-liquid phase split. Below, we discuss the impact of each hyperparameter in detail.

Impact of Gibbs loss

The purpose of the Gibbs loss is to force the model to produce correct Δg_{mix} curves that lead to a phase split for the LLE data in the training. The loss is applied only to data points for which this is not the case (cf. Section "Training of HANNA" in the manuscript) and should therefore have little impact on the other data types. In our preliminary studies, incorporating the Gibbs loss into the training increased the number of LLE phase splits that HANNA could correctly predict, thereby expanding the model's applicability range. We noticed that while high values of w_{Gibbs} lead to a larger number of systems with correctly predicted phase splits, they also yield two-phase regions that extend beyond the experimentally measured upper and lower critical solution temperatures. This is likely because, through this "conservative" behavior, the model avoids the Gibbs loss. Consequently, we selected $w_{\text{Gibbs}} = 0.001$ as a good compromise for the final model configuration.

Impact of Lipschitz Regularization

Lipschitz regularization controls the smoothness of neural network outputs by encouraging a small Lipschitz constant. A higher regularization weight w_{Lips} promotes more smoothness but restricts the flexibility of the

model. The ideal regularization weight should still provide the model with sufficient flexibility to extrapolate to unseen data. In our preliminary studies, for smaller values of w_{Lips}, we found that the number of LLE systems with unphysical artifacts, such as sudden changes in phase compositions over temperature, increased. On the other hand, larger values of w_{Lips} led to a higher error on the ACI data. We decided to prioritize model smoothness and robustness over very high accuracy on ACI data and chose $w_{\text{Lips}} = 0.0005$ for the final model configuration.

Impact of TPX training

In Suppl. Fig. 2, the impact of the TPX weight w_{TPX} on the model accuracy over different data types is shown. As expected, increasing w_{TPX} leads to a lower error on the TPX data. For $w_{\text{TPX}} = 2.0$, a slight decrease in accuracy on ACI data is visible. For all other data types, the TPX data weight during training appears to have no significant impact. For the final model configuration, we therefore chose $w_{\text{TPX}} = 1.0$.

Impact of LLE training

Suppl. Fig. 3 shows how the training on LLE data impacts the model accuracy on the different data types. The validation scores on the TPXY, TPX, and HE data do not change significantly with varying LLE loss weights, whereas the errors on the LLE data are greatly reduced by increasing w_{LLE}. The percentage of correctly predicted phase splits slightly improves with increasing w_{LLE}. This shows that training on LLE data enables accurate predictions for unseen LLE systems and, more importantly, that the surrogate solver works as intended during training. High values of w_{LLE} lead to higher errors on the ACI data. Based on the grid search result, we selected $w_{\text{LLE}} = 2.0$ for the final model configuration.

Impact of HE training

The impact of the training on HE data on the model accuracy on different data types is shown in Suppl. Fig. 4. For the model variants trained without HE data (i.e., $w_{\text{HE}} = 0.0$), the validation loss on HE data is extremely high. Apparently, the other data types (TPXY, TPX, ACI, and LLE) do not provide sufficient information on the temperature derivative of the excess Gibbs energy for the model to be able to accurately describe excess enthalpies. As expected, increasing w_{HE} leads to a significant improvement in the accuracy on the HE data. On the TPXY and TPX data, we see little to no impact of the training on the HE data. For ACI and LLE data, we observe a slight decline in accuracy. The share of correctly predicted phase splits also decreases for higher values of w_{HE}. Because the accurate prediction of activity coefficients is the main focus of this work, we chose $w_{\text{HE}} = 0.5$ for our final model configuration.

Supplementary Table 1: Hyperparameter search space used in the grid search. The hyperparameter values of the model configuration selected for training the final models are denoted in bold.

Hyperparameter	Search space
w_{Lips}	$\mathbf{5 \cdot 10^{-4}}$
w_{Gibbs}	$\mathbf{0.001}$
w_{TPX}	$0.0, 0.5, \mathbf{1.0}, 2.0$
w_{LLE}	$0.0, \mathbf{2.0}, 5.0, 10.0, 20.0$
w_{HE}	$0.0, \mathbf{0.5}, 1.0, 2.0$

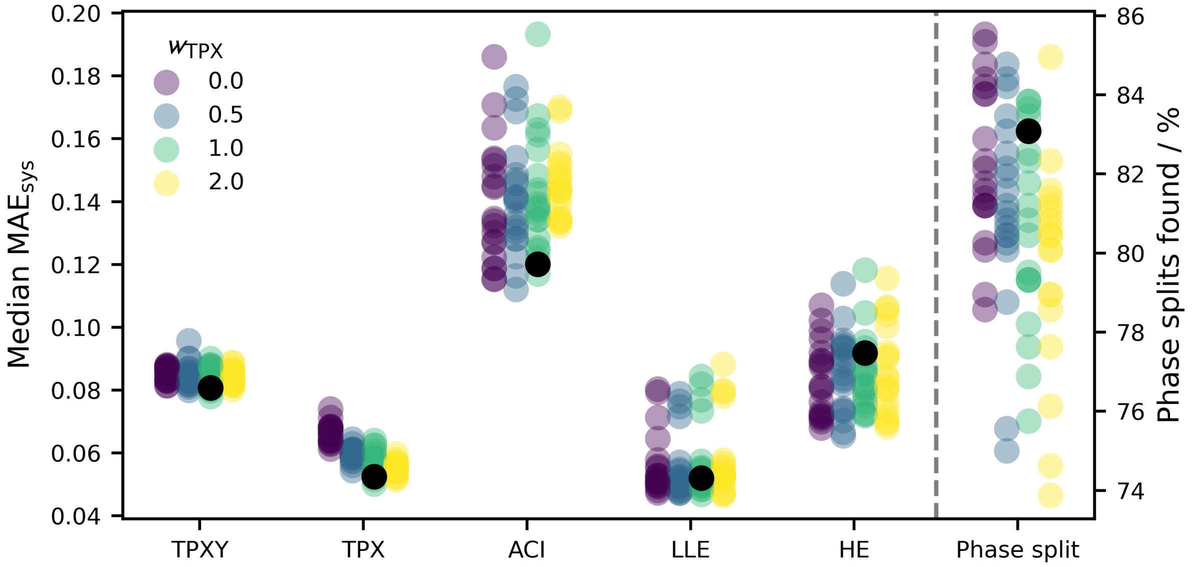

Supplementary Figure 2: Scatter plot visualizing the impact of different weights w_{TPX} of the TPX loss term $\mathcal{L}_{\mathrm{TPX}}$ on the model accuracy on validation data for the five different data types. For each data type, all models from the grid search are colored and aligned by their TPX loss weight during training. The group on the outer right shows the percentage of systems for which a liquid-liquid phase split is correctly predicted. The black markers denote the selected model configuration.

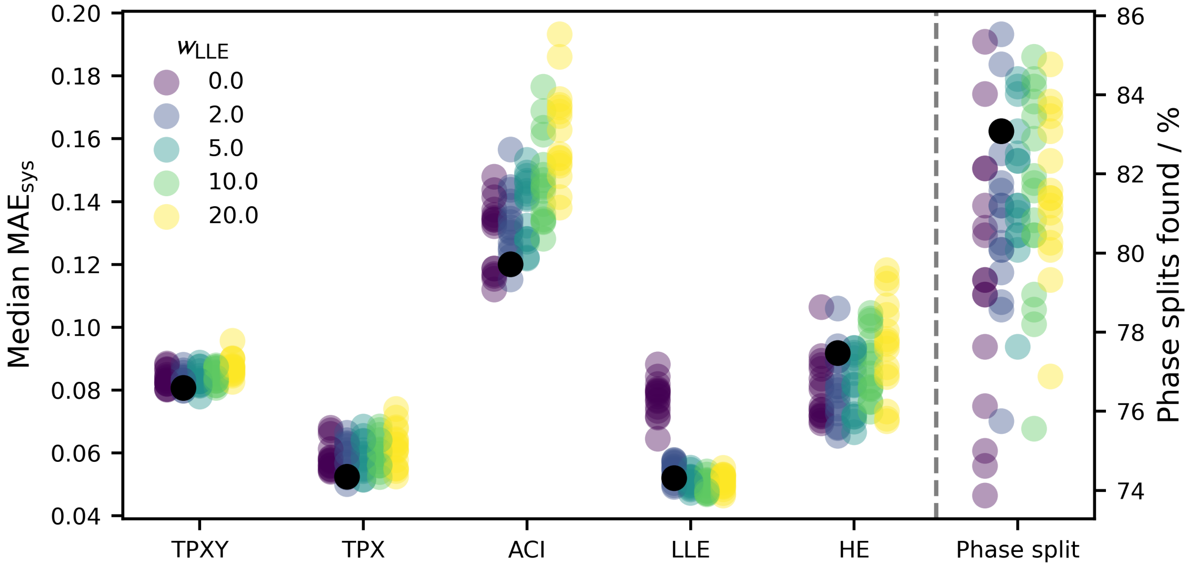

Supplementary Figure 3: Scatter plot visualizing the impact of different weights w_{LLE} of the LLE loss $\mathcal{L}_{\mathrm{LLE}}$ on the model accuracy on validation data for the five different data types. For each data type, all models from the grid search are colored and aligned by their w_{LLE} during training. The group on the outer right shows the percentage of systems for which a liquid-liquid phase split is correctly predicted. The black markers denote the selected model configuration.

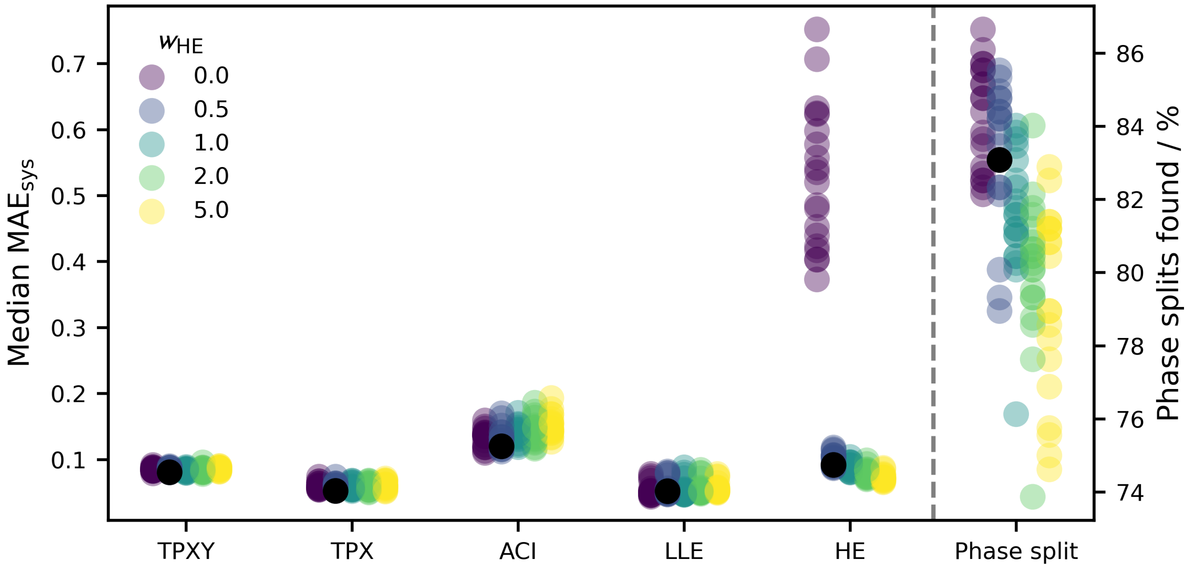

Supplementary Figure 4: Scatter plot visualizing the impact of different weights w_{HE} of the HE loss $\mathcal{L}_{\mathrm{HE}}$ on the model accuracy on validation data for the three different data types. For each data type, all models from the grid search are colored and aligned by their HE loss weights during training. The group on the outer right shows the percentage of systems for which a liquid-liquid phase split is correctly predicted. The black markers denote the selected model configuration.

Suppl. Note 3. Additional results

3.1 Comparison to other ML models

Supplementary Table 2 compares HANNA with different ML models for predicting activity coefficients, assessing their range of applicability and their consistency with thermodynamic constraints. All literature models are limited to predictions at 298 K; only HANNA is capable of predicting temperature-dependent activity coefficients. Moreover, HANNA is the only model that is consistent in the pure components ($\gamma_i \rightarrow 1$ for $x_i \rightarrow 1$) and models pseudo-mixtures composed of the same components as pure components through its hard-constraint architecture. Aside from SolvGNN, which is also applicable to ternary systems, all literature models are restricted to binary systems.

Supplementary Table 2: Comparison of HANNA with different ML models from the literature for the prediction of activity coefficients regarding their applicability and strict compliance with consistency criteria. Here, only disclosed models that are not based on the equation framework of physics-based g^{E}-models are considered. Consistency criteria are (cf. discussion above): Gibbs-Duhem consistency (GD), permutation equivariance (PE), consistency in the pure components (PC), consistency for pseudo-mixtures (PM).

	**Applicability**				**Consistency**			
Model	γ^∞ at 298 K	$\gamma_i(x_i)$ at 298 K	$\gamma_i(x_i, T)$	Ternary systems	GD	PE	PC	PM
HANNA	✓	✓	✓	✓	✓	✓	✓	✓
GE-GNN[2]	✓	✓	✗	✗	✓	✓	✗	✗
GDI-GNN[3]	✓	✓	✗	✗	✗	✓	✗	✗
SolvGNN[4]	✓	✓	✗	✓	✗	✓	✗	✗
GNN-IAC[5]	✓	✗	✗	✗	–[1]	–[1]	–[1]	✗

[1]Because GNN-IAC is restricted to predicting activity coefficients at infinite dilution, the consistency criteria do not apply here.

3.2 Three-phase and island type ternary LLE

In Fig. 3c in the manuscript, we demonstrate that HANNA can successfully predict LLE in ternary systems with one (top) and two (bottom) binary subsystems that exhibit a miscibility gap. In Suppl. Fig. 5, we additionally show predictions of HANNA for LLE in ternary systems with zero and three such binary subsystems. The two systems at the top are of type III in the Treybal classification [6], i.e., for some compositions they form a three-phase equilibrium at the considered temperature. For the shown systems, HANNA correctly predicts the three-phase region, with relatively good agreement between the predicted phase compositions and experimental test data. The systems shown at the bottom of Suppl. Fig. 5 exhibit an island-type behavior. While the binary subsystems are completely miscible, a miscibility gap appears at certain ternary compositions. For these systems, HANNA qualitatively predicts the correct behavior, but the predicted phase compositions deviate from the experimental data.

3.3 Predictions for the HE data

In Suppl. Fig. 6, the performance of HANNA for the prediction of excess enthalpies is evaluated. The boxplots in Suppl. Fig. 6a compare the prediction accuracy of HANNA with that of mod. UNIFAC on both the mod. UNIFAC horizon and the full horizon for binary (top) and ternary (bottom) systems. The results show superior accuracy for HANNA in both cases. In Suppl. Fig. 6b, exemplary results for four binary systems are shown. In contrast to all other investigated data types (TPXY, TPX, ACI, LLE), which mainly rely on derivatives of g^{E} w.r.t. composition, the prediction of excess enthalpies is directly dependent on the temperature derivatives of g^{E}. The results in Suppl. Fig. 6 demonstrate that HANNA can correctly model the temperature derivative of the excess Gibbs energy and, by extension, the excess enthalpy for previously unseen systems. Based on the results of the grid search in Suppl. Fig. 4, it is evident that the direct training on HE data is required to achieve this.

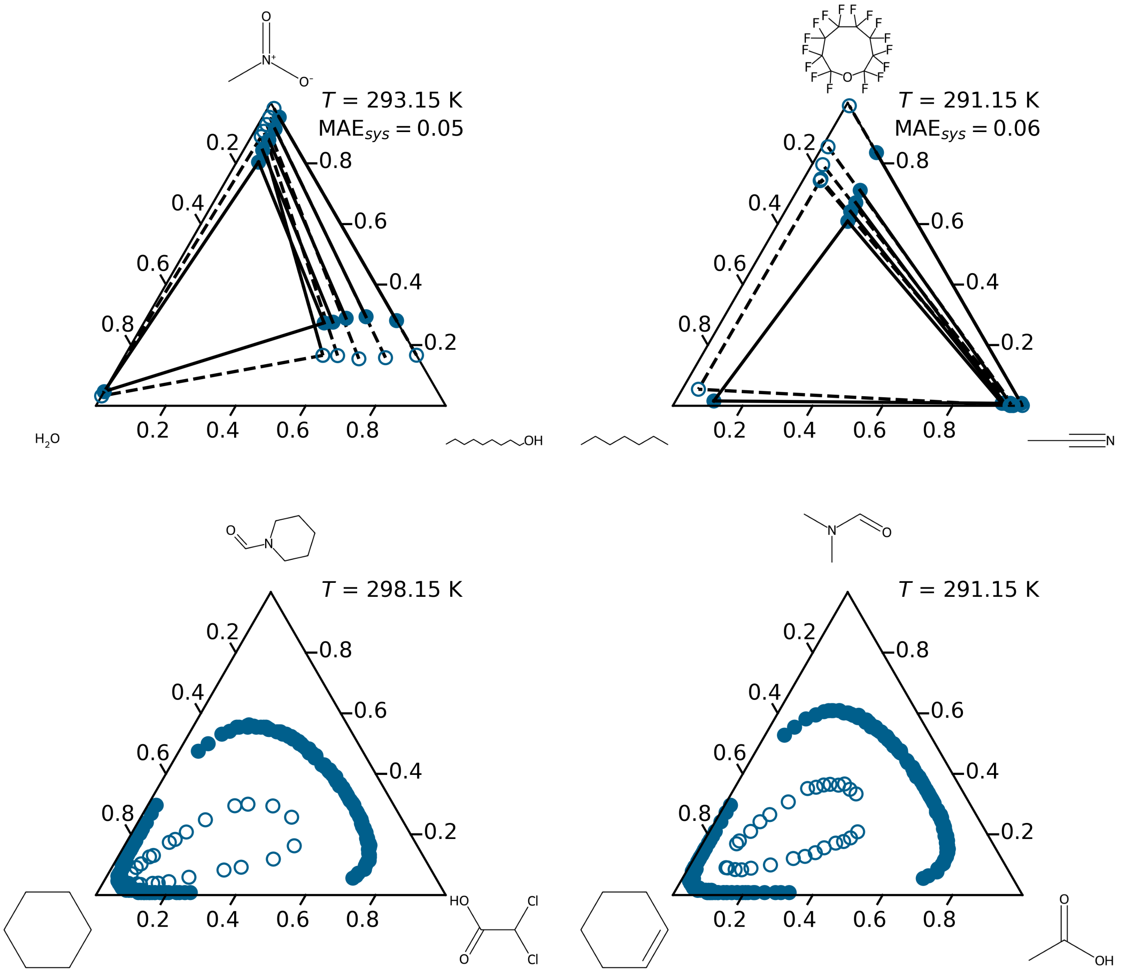

Supplementary Figure 5: Predictions of HANNA for isothermal LLE in ternary systems. Top: Systems with three liquid phases in equilibrium; data from the DDB [7]. Bottom: Systems with an island-type behavior (no miscibility gaps in the binary subsystems); data taken from Ref. [8]. Open symbols and dashed tie lines represent the experimental data; filled symbols and solid lines represent predictions by HANNA. Note that for the island-type systems a MAE_{sys} cannot be calculated, because only one phase composition is given per data point, such that the feed composition is unknown.

3.4 Predictions for the TPX data

In Suppl. Fig. 7, the performance of HANNA for the prediction of total pressures in VLE (for the TPX data) is evaluated. The boxplots in Suppl. Fig. 7a compare the prediction accuracy of HANNA with that of mod. UNIFAC on both the mod. UNIFAC horizon and the full horizon for binary (top) and ternary (bottom) systems. For binary systems, HANNA performs slightly better than mod. UNIFAC. For ternary systems, both methods work equally well. On the full horizon for ternary systems, the prediction accuracy of HANNA declines.

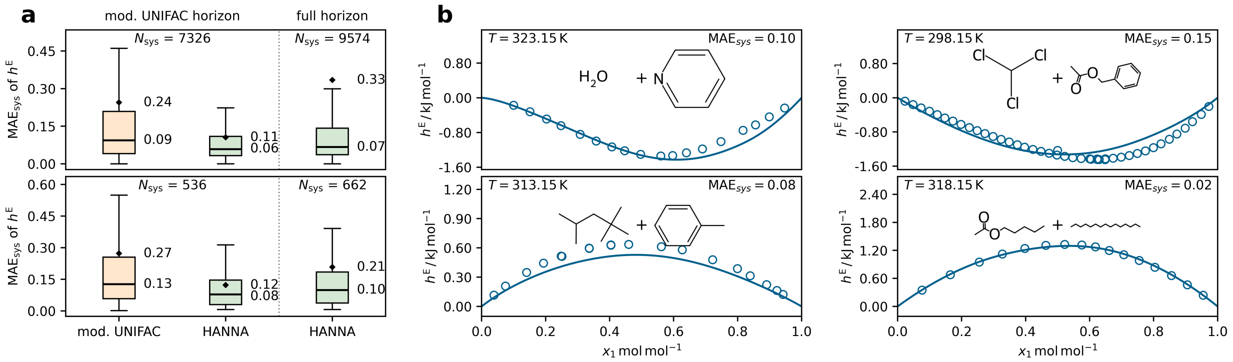

Supplementary Figure 6: Predictions for excess enthalpies h^E with HANNA. **a**, Boxplots comparing the performance of HANNA for predicting h^E in binary (top) and ternary (bottom) systems with that of mod. UNIFAC in terms of the system-wise mean absolute error MAE_{sys}. For a fair comparison, HANNA was also evaluated only on those systems for which mod. UNIFAC is applicable (mod. UNIFAC horizon). N_{sys} denotes the number of test systems within the respective horizon. The boxes represent interquartile ranges, and the whiskers are 1.5 times the interquartile range. Diamonds mark the mean, horizontal lines the median of the MAE_{sys} values. **b**, Predictions of HANNA for h^E of four binary systems compared to experimental data. Open symbols denote experimental data, lines are predictions by HANNA. The molecular structures of the components and the respective MAE_{sys} are depicted in the plots; the left molecule corresponds to component 1.

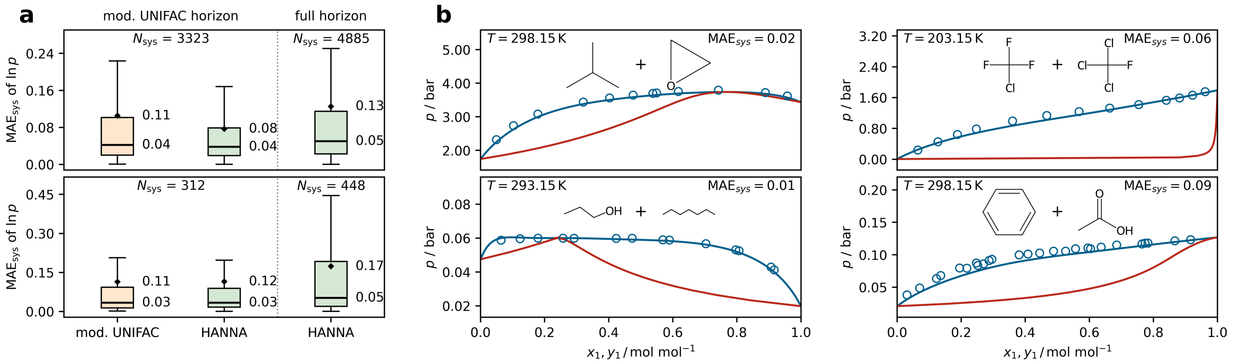

Supplementary Figure 7: Predictions for TPX data with HANNA. **a**, Boxplots comparing the performance of HANNA for predicting the total pressures in binary (top) and ternary (bottom) systems in VLE with that of mod. UNIFAC in terms of the system-wise mean absolute error MAE_{sys} in $\ln p$. For a fair comparison, HANNA was also evaluated only on those systems for which mod. UNIFAC is applicable (mod. UNIFAC horizon). N_{sys} denotes the number of test systems within the respective horizon. The boxes represent interquartile ranges, and the whiskers are 1.5 times the interquartile range. Diamonds mark the mean, horizontal lines the median of the MAE_{sys} values. **b**, Predictions of HANNA for the total pressures (calculated through extended Raoult's law) in four binary systems plotted against experimental data. Open symbols denote experimental data, and blue lines are predictions by HANNA. For convenience, the corresponding predicted dew lines are shown as red lines. The molecular structures of the components and the respective MAE_{sys} are depicted in the plots; the left molecule corresponds to component 1.

3.5 Binary phase equilibria with mod. UNIFAC

Suppl. Fig. 8 shows the phase equilibria for six binary systems predicted with HANNA and mod. UNIFAC. The systems correspond to those in Fig. 2b, 2c, and 2d of the manuscript that are within the mod. UNIFAC horizon, i.e., two systems in VLE (left column, top and middle), one system in LLE (left column, bottom), and three heteroazeotropes (right column). Note that mod. UNIFAC was likely fitted to the systems presented here, and the comparison is therefore biased towards mod. UNIFAC.

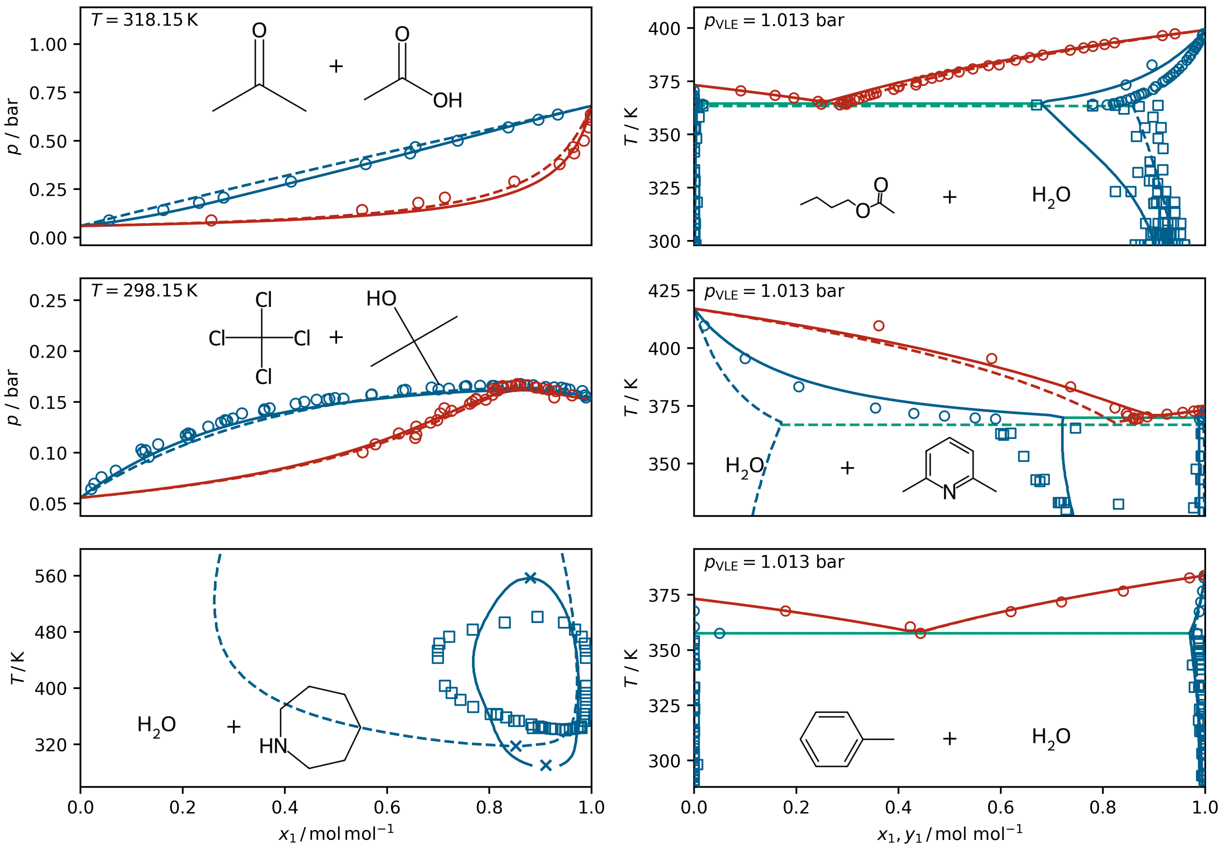

Supplementary Figure 8: Predicted phase equilibria with HANNA (solid lines) and mod. UNIFAC (dashed lines) for six binary systems. Open blue and red circles indicate the experimental liquid and vapor phases, respectively, from VLE data. Open blue squares mark the experimental phase compositions from LLE data. The green lines are the predicted vapor-liquid-liquid equilibria. The molecular structures of the components are depicted in the plots; the left molecule corresponds to component 1.

3.6 Predictions for quaternary systems

Suppl. Fig. 9 compares the TPXY and LLE predictions of HANNA for quaternary systems to those of mod. UNIFAC. Their performance is similar on the shared horizon of both data types. On the full horizon, the accuracy of HANNA declines.

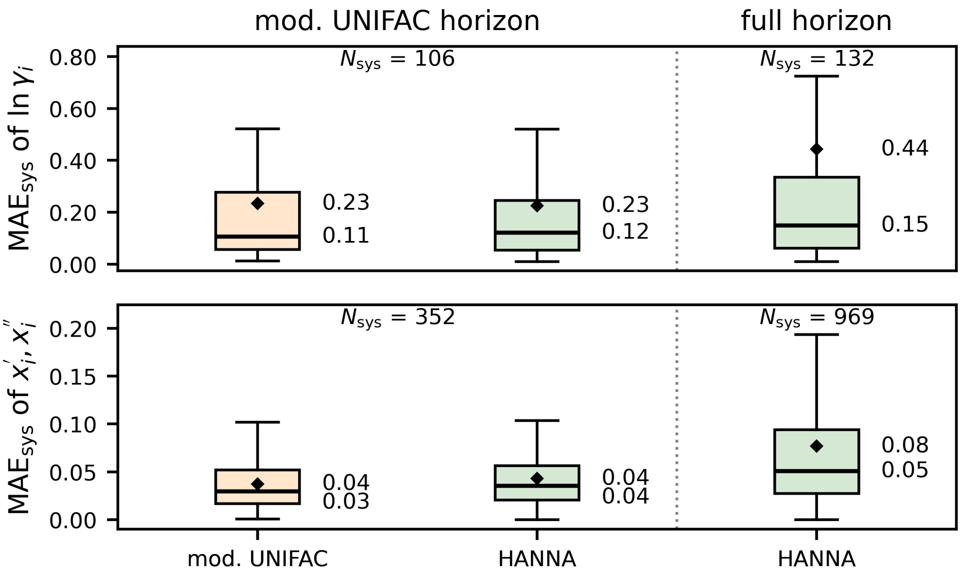

Supplementary Figure 9: Boxplots comparing the performance of HANNA for predicting activity coefficients in quaternary VLE (TPXY data, top) and LLE phase compositions (bottom) with that of mod. UNIFAC in terms of the system-wise mean absolute error $\mathrm{MAE_{sys}}$ in $\ln \gamma_i$ (TPXY) or phase compositions x_i' and x_i'' (LLE). For a fair comparison, HANNA was also evaluated only on those systems for which mod. UNIFAC is applicable (mod. UNIFAC horizon). N_{sys} denotes the number of test systems within the respective horizon and data type. The boxes represent interquartile ranges, and the whiskers are 1.5 times the interquartile range. Diamonds mark the mean, horizontal lines the median of the $\mathrm{MAE_{sys}}$ values.

3.7 Predictions for systems containing ionic liquids

Suppl. Fig. 10 compares the ACI and LLE predictions of HANNA for systems containing ionic liquids to those of mod. UNIFAC. A system was selected only if the SMILES of at least one of the components contained the following characters: '+' (positive charge) corresponding to the cation; '-' (negative charge) corresponding to the anion; '.' (the SMILES notation of the "non-bond" that separates the cation and anion).

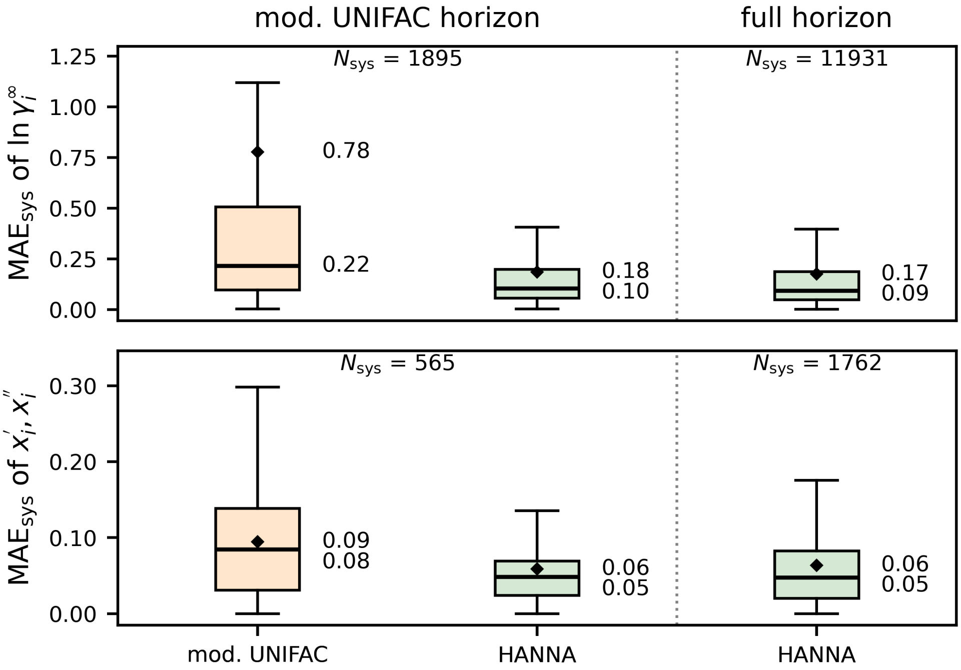

Supplementary Figure 10: Boxplots comparing the performance of HANNA for predicting binary ACI (top), and LLE phase compositions (bottom) that include at least one IL component with that of mod. UNIFAC in terms of the system-wise mean absolute error $\mathrm{MAE_{sys}}$ in γ_i^∞ (ACI) or phase compositions x_i' and x_i'' (LLE). For a fair comparison, HANNA was also evaluated only on those systems for which mod. UNIFAC is applicable (mod. UNIFAC horizon). N_{sys} denotes the number of test systems within the respective horizon and data type. The boxes represent interquartile ranges, and the whiskers are 1.5 times the interquartile range. Diamonds mark the mean, horizontal lines the median of the $\mathrm{MAE_{sys}}$ values.

3.8 Comparison of HANNA to mod. UNIFAC 2.0

In Suppl. Figs. 11, 12, 13, HANNA is compared to mod. UNIFAC 2.0 [9] for binary, ternary, and quaternary systems, respectively. Note that the training of mod. UNIFAC 2.0 included HE data but did not include any LLE data.

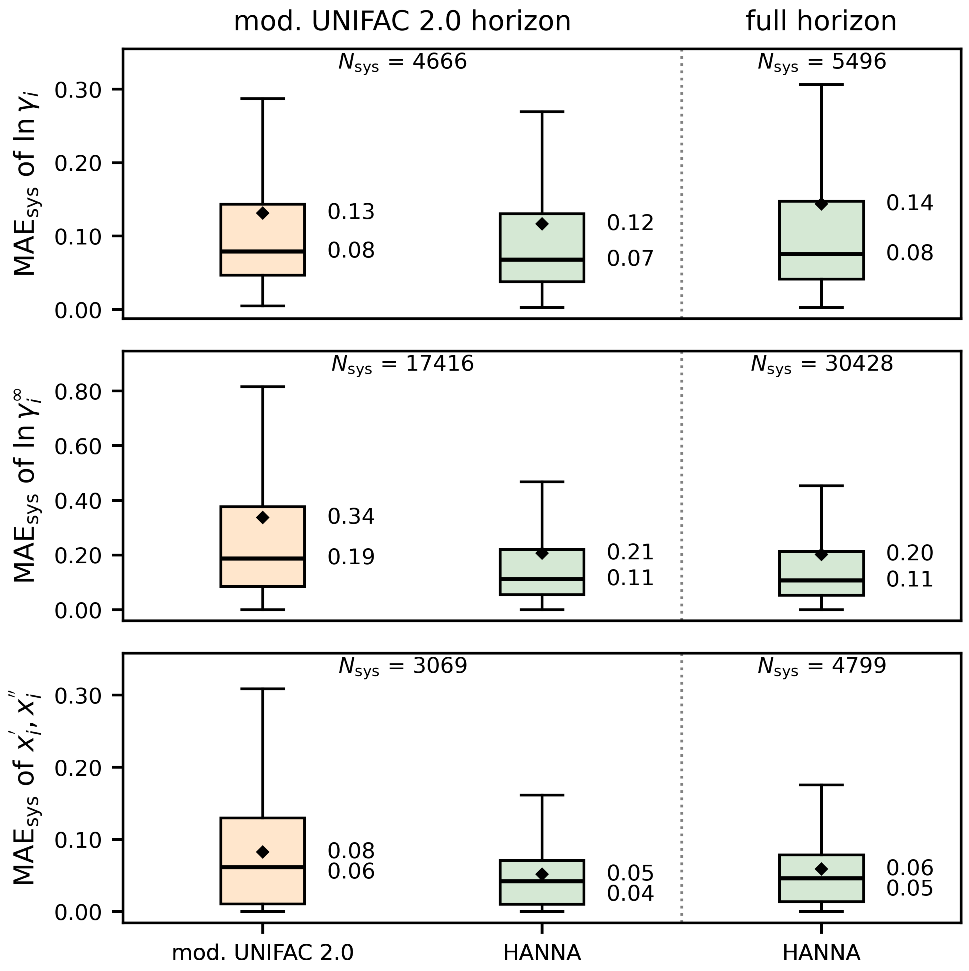

Supplementary Figure 11: Boxplots comparing the performance of HANNA for predicting activity coefficients from VLE (TPXY data, top), ACI (middle), and LLE phase compositions (bottom) in binary systems with that of mod. UNIFAC 2.0 [9] in terms of the system-wise mean absolute error MAE$_{\text{sys}}$ in $\ln \gamma_i$ (VLE, ACI) or phase compositions x_i' and x_i'' (LLE). For a fair comparison, HANNA was also evaluated only on those systems for which mod. UNIFAC 2.0 is applicable (mod. UNIFAC 2.0 horizon). N_{sys} denotes the number of test systems within the respective horizon and data type. The boxes represent interquartile ranges, and the whiskers are 1.5 times the interquartile range. Diamonds mark the mean, horizontal lines the median of the MAE$_{\text{sys}}$ values.

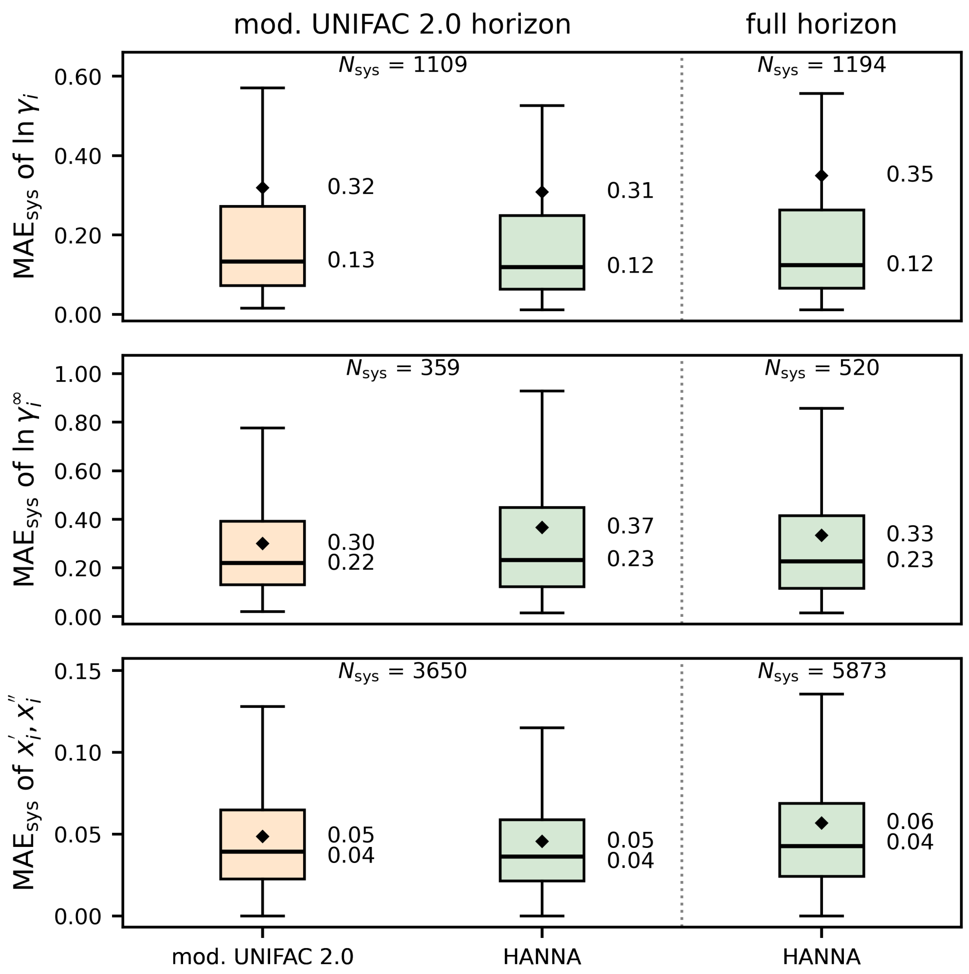

Supplementary Figure 12: Boxplots comparing the performance of HANNA for predicting activity coefficients from VLE (TPXY data, top), ACI (middle), and LLE phase compositions (bottom) in ternary systems with that of mod. UNIFAC 2.0 [9] in terms of the system-wise mean absolute error $\mathrm{MAE}_{\mathrm{sys}}$ in $\ln \gamma_i$ (VLE, ACI) or phase compositions x_i' and x_i'' (LLE). For a fair comparison, HANNA was also evaluated only on those systems for which mod. UNIFAC 2.0 is applicable (mod. UNIFAC 2.0 horizon). N_{sys} denotes the number of test systems within the respective horizon and data type. The boxes represent interquartile ranges, and the whiskers are 1.5 times the interquartile range. Diamonds mark the mean, horizontal lines the median of the $\mathrm{MAE}_{\mathrm{sys}}$ values.

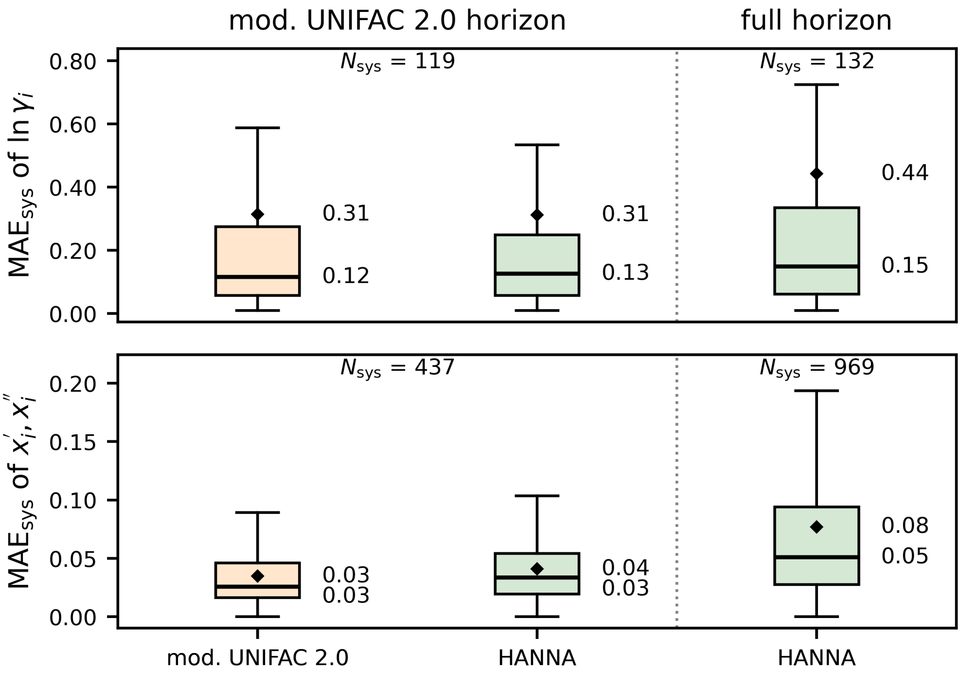

Supplementary Figure 13: Boxplots comparing the performance of HANNA for predicting activity coefficients from VLE (TPXY data, top) and LLE phase compositions (bottom) in quaternary systems with that of mod. UNIFAC 2.0 [9] in terms of the system-wise mean absolute error $\mathrm{MAE_{sys}}$ in $\ln \gamma_i$ (VLE) or phase compositions x_i' and x_i'' (LLE). For a fair comparison, HANNA was also evaluated only on those systems for which mod. UNIFAC 2.0 is applicable (mod. UNIFAC 2.0 horizon). N_{sys} denotes the number of test systems within the respective horizon and data type. The boxes represent interquartile ranges, and the whiskers are 1.5 times the interquartile range. Diamonds mark the mean, horizontal lines the median of the $\mathrm{MAE_{sys}}$ values.

3.9 Comparison of HANNA to UNIFAC-LLE

In Suppl. Fig. 14, HANNA is compared to UNIFAC-LLE [10] on binary, ternary, and quaternary LLE data, respectively. Note that UNIFAC-LLE is a parameterization that is only designed for the prediction of LLE in the temperature range between 283.15 K and 313.15 K, and therefore no comparison on the VLE, ACI, or HE data sets was performed. Furthermore, the comparison on the UNIFAC-LLE horizon includes only data points within the respective temperature range. Note that UNIFAC-LLE included ternary LLE data in the training, resulting in a positive bias for ternary and quaternary data.

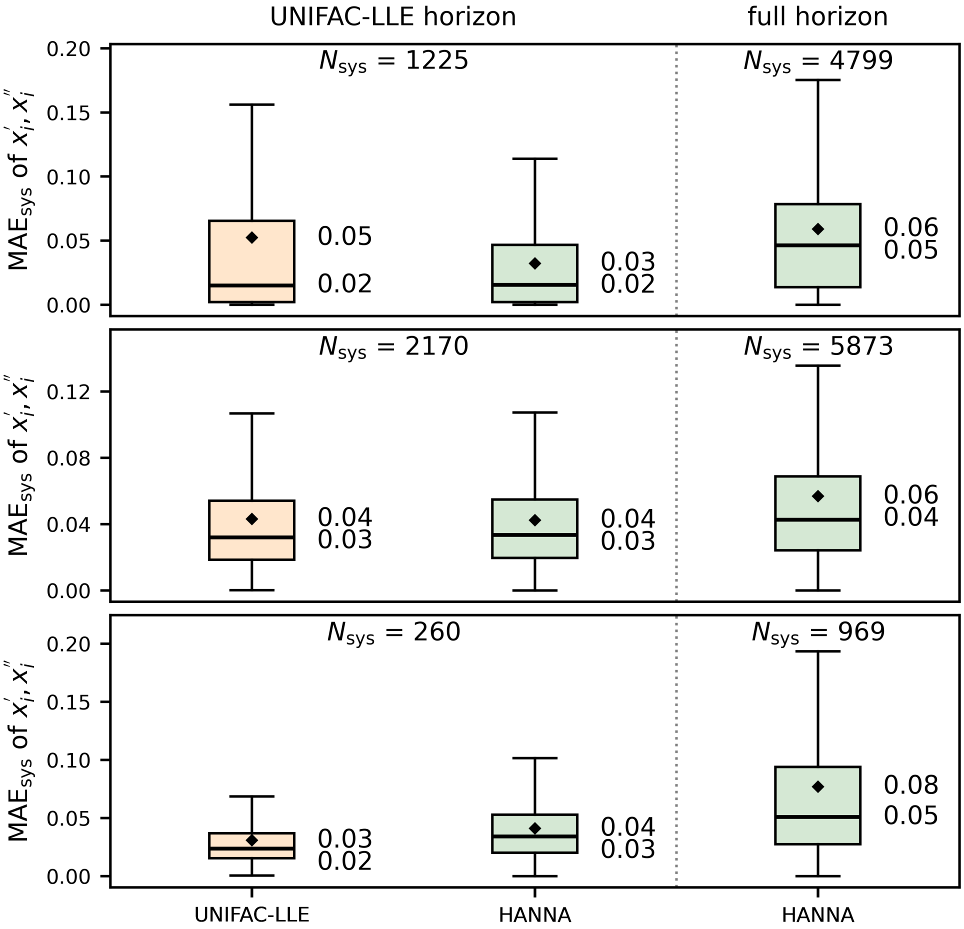

Supplementary Figure 14: Boxplots comparing the performance of HANNA for predicting binary (top), ternary (middle), and quaternary (bottom) LLE phase compositions with that of UNIFAC-LLE [10] in terms of the system-wise mean absolute error $\mathrm{MAE}_{\mathrm{sys}}$ in x_i' and x_i''. For a fair comparison, HANNA was also evaluated only on those systems and in the temperature range (283.15 K and 313.15 K) for which UNIFAC-LLE is applicable (UNIFAC-LLE horizon). N_{sys} denotes the number of test systems within the respective horizon and data type. The boxes represent interquartile ranges, and the whiskers are 1.5 times the interquartile range. Diamonds mark the mean, horizontal lines the median of the $\mathrm{MAE}_{\mathrm{sys}}$ values.

Suppl. Note 4.

Evaluation of the projection quality to multi-component systems

Suppl. Figs. 15 and 16 show a comparison of the extrapolation capabilities of HANNA using the geometric projection method of Muggianu [11] to the extrapolation of the most used g^{E} models UNIQUAC [12] and NRTL [13] on ternary TPXY and ACI data. For this purpose, we fitted the respective parameters of the g^{E} models ($\Delta u_{ij}, \Delta u_{ij}$ for UNIQUAC; α, A_{ij}, A_{ji} for NRTL) for each binary subsystem of a ternary system on the predictions of HANNA. This approach ensures that we have an identical description of the ternary system in the binary subsystems and `only` compare the differences arising from their extrapolation behavior going from binary to ternary systems. We found that our simple geometric method shows better or comparable extrapolation performance to both UNIQUAC and NRTL in the considered systems.

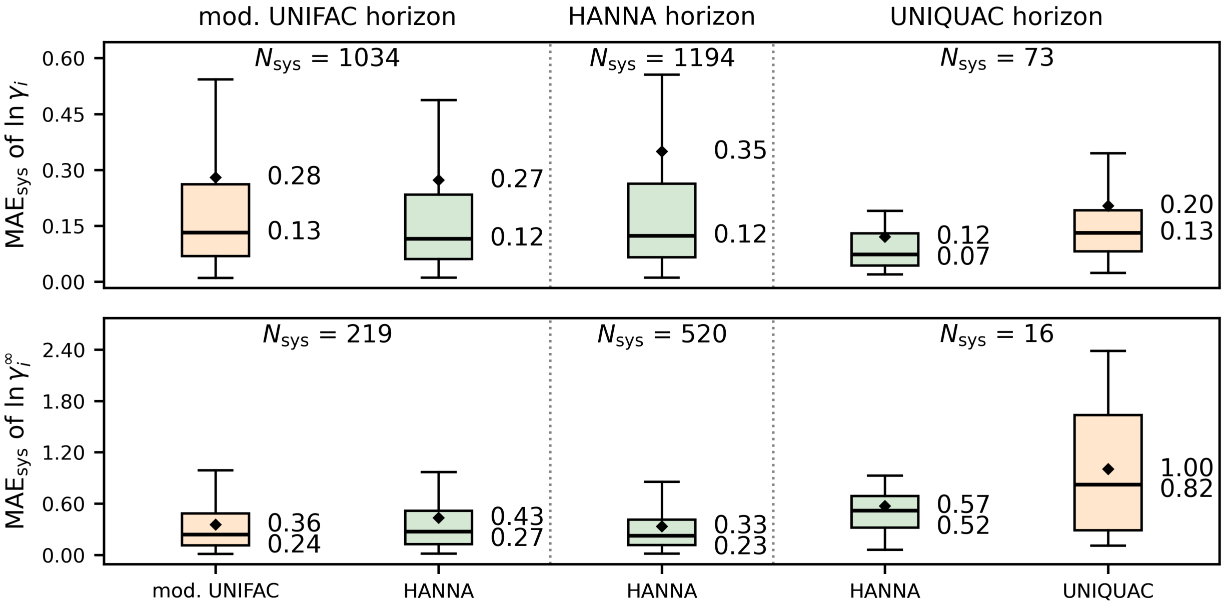

Supplementary Figure 15: Boxplots comparing the accuracy of HANNA (and mod. UNIFAC) in extrapolating to activity coefficients in ternary systems derived from VLE (TPXY) data (top) and ACI data (bottom) with that of UNIQUAC [12]. The UNIQUAC predictions for a ternary system were obtained by first fitting the parameters Δu_{ij} and Δu_{ji} of the three binary subsystems to predictions of the logarithmic activity coefficients with HANNA. The UNIQUAC horizon only comprises systems for which this fit succeeded and the mean deviation of $\ln \gamma_i$ lies below 15 %. N_{sys} denotes the number of test systems within the respective horizon and data type. The boxes represent interquartile ranges, and the whiskers are 1.5 times the interquartile range. Diamonds mark the mean, horizontal lines the median of the $\mathrm{MAE_{sys}}$ values.

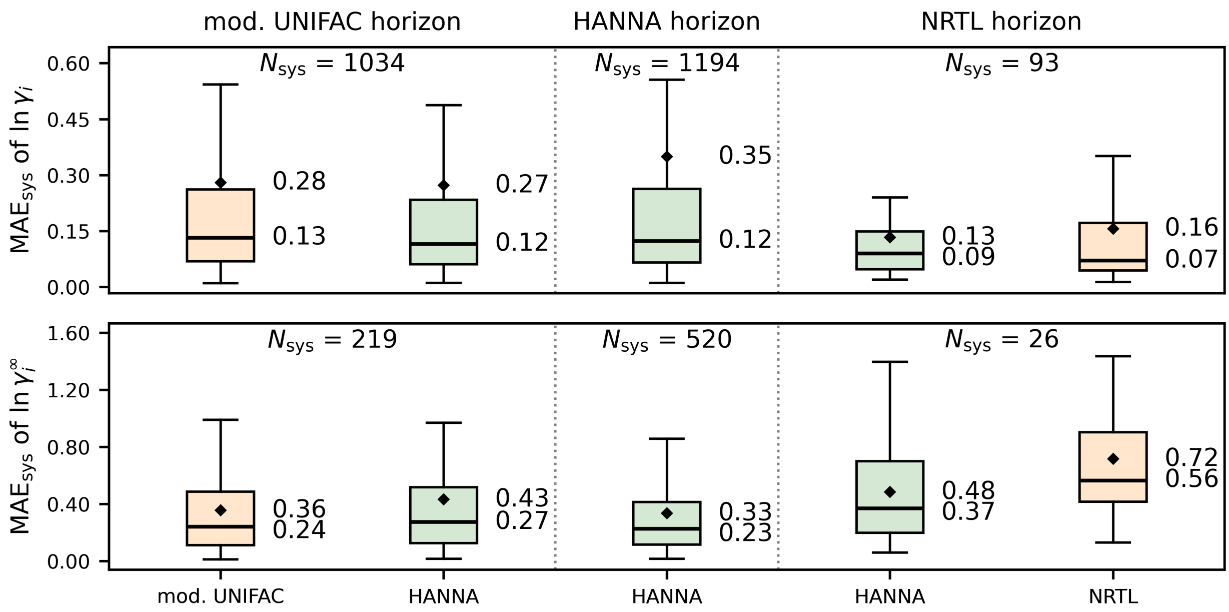

Supplementary Figure 16: Boxplots comparing the accuracy of HANNA (and mod. UNIFAC) in extrapolating to activity coefficients in ternary systems derived from VLE (TPXY) data (top) and ACI data (bottom) with that of NRTL [13]. The NRTL predictions for a ternary system were obtained by first fitting the parameters Δu_{ij} and Δu_{ji} of the three binary subsystems to predictions of the logarithmic activity coefficients with HANNA. The NRTL horizon only comprises systems for which this fit succeeded and the mean deviation of $\ln \gamma_i$ lies below 15 %. $N_{\rm sys}$ denotes the number of test systems within the respective horizon and data type. The boxes represent interquartile ranges, and the whiskers are 1.5 times the interquartile range. Diamonds mark the mean, horizontal lines the median of the $\mathrm{MAE_{sys}}$ values.

Supplementary references

[1] Specht, T. *et al.* HANNA: Hard-constraint neural network for consistent activity coefficient prediction. *Chemical Science* **15**, 19777–19786 (2024).

[2] Rittig, J. G. & Mitsos, A. Thermodynamics-consistent graph neural networks. *Chemical Science* **15**, 18504–18512 (2024).

[3] Rittig, J. G., Felton, K. C., Lapkin, A. A. & Mitsos, A. Gibbs–duhem-informed neural networks for binary activity coefficient prediction. *Digital Discovery* **2**, 1752–1767 (2023).

[4] Qin, S. *et al.* Capturing molecular interactions in graph neural networks: a case study in multi-component phase equilibrium. *Digital Discovery* **2**, 138–151 (2023).

[5] Sanchez Medina, E. I., Linke, S., Stoll, M. & Sundmacher, K. Graph neural networks for the prediction of infinite dilution activity coefficients. *Digital Discovery* **1**, 216–225 (2022).

[6] Treybal, R. *Liquid extraction* (McGraw-Hill, 1963).

[7] Dortmund Data Bank. https://www.ddbst.com (2025).

[8] Becker, F. & Richter, P. Non-aqueous ternary mixtures with 'island' miscibility gaps. *Fluid Phase Equilibria* **49**, 157–166 (1989).

[9] Hayer, N., Hasse, H. & Jirasek, F. Modified UNIFAC 2.0-a group-contribution method completed with machine learning. *Industrial & Engineering Chemistry Research* **64**, 10304–10313 (2025).

[10] Magnussen, T., Rasmussen, P. & Fredenslund, A. UNIFAC parameter table for prediction of liquid-liquid equilibriums. *Industrial & Engineering Chemistry Process Design and Development* **20**, 331–339 (1981).

[11] Muggianu, Y.-M., Gambino, M. & Bros, J.-P. Enthalpies de formation des alliages liquides bismuth-étain-gallium à 723 k. choix d'une représentation analytique des grandeurs d'excès intégrales et partielles de mélange. *Journal de Chimie Physique* **72**, 83–88 (1975).

[12] Abrams, D. S. & Prausnitz, J. M. Statistical thermodynamics of liquid mixtures: a new expression for the excess gibbs energy of partly or completely miscible systems. *AIChE Journal* **21**, 116–128 (1975).

[13] Renon, H. & Prausnitz, J. M. Local compositions in thermodynamic excess functions for liquid mixtures. *AIChE Journal* **14**, 135–144 (1968).
